# Surface plasmon induced spot and line formation at interfaces of ITO coated LiNbO_3_ slabs and gigantic nonlinearity

**DOI:** 10.1038/s41598-021-99270-4

**Published:** 2021-10-05

**Authors:** Zuoren Xiong, Xinyan Ma, Yanbo Pei, Yingbin Zhang, Hua Zhao

**Affiliations:** 1 Institute of Modern Optics, School of Physics, Harbin Institute of Technology, Harbin, 150001 China; 2 Key Laboratory of Micro-Optics and Photonics Technology of Heilongjiang Province, Harbin, 150001 China; 3 Key Laboratory of Micro-Nano Optoelectronic Information System, Ministry of Industry and Information Technology, Harbin, 150001 China

**Keywords:** Plasma physics, Nonlinear optics

## Abstract

Remarkable spots and lines were clearly observed at the two interfaces of indium-tin-oxide coated Z-cut Fe-doped lithium noibate plates under illumination by milliwatt continuous-wave laser light; this occurred because of the visible surface plasmons (SPs) supported by the promising non-metal plasmonic system. The intriguing observations are here explained via the SP-strengthened nonlinear effect, through consideration of the electrostatic field (which is comparable to the atomic field) and its large gradient; this hints at a promising, highly sensitive plasmonic system. The gigantic nonlinear effect discussed in this paper should be ubiquitously existed in many oxide ferroelectric/semiconductor combinations and is promising for visible plasmonic applications.

## Introduction

Plasmonics is a well-established, rapidly expanding field that has stimulated the development of a variety of exotic devices and concepts, owing to the confinement of electromagnetic energy associated with the evanescent nature of surface plasmons (SPs)^1–4^. To bypass the challenges associated with large-loss metal usage, alternative plasmonic materials have recently become a research hotspot^5,6^. Facilitated by the nanometric fabrication of materials and their heterostructures, near-perfect interfaces can be routinely prepared, resulting in intriguing emergent phenomena^7^, including the metallization of surfaces^8^. Because SP excitations generate intensified electromagnetic fields, nonlinear optical effects can be considerably enhanced when a nonlinear optical material is coated with transparent conducting film^9,10^. The nonlinear effects can be remarkably enhanced by using gratings to excite SPs^11,12^. To study the influence of SPs on the nonlinearity of optical materials and tailor their optical responses, indium-tin-oxide (ITO) films were deposited onto slabs of Fe-doped lithium noibate (Fe:LiNbO_3_; Fe:LN) synthetic crystals; these are among the most investigated and commercially popular nonlinear optical materials, and they continue to attract considerable attention^13^.

In the recent past, interesting results have been found and reported for ITO-coated LN slabs^13^. Visible SPs can be supported in LN systems, owing to the 2D electron gas (2DEG) formed at interfaces and surfaces; however, an in-depth understanding of what occurs remains elusive in a deeper, broader sense^14^. For instance, highly efficient SPs producing nonlinear effects have been neglected^11,12^. More specifically, 2DGEs are formed adjacent to highly nonlinear material LNs; this can support visible SPs. In addition, near to the 2DEG layer is an ultra-high depolarization field that is comparable to an atomic field and features a large field gradient. Under such conditions, striking nonlinear effects are manifested. Using a microscopic system to monitor the dynamics near the two ITO/LN interfaces, we observed singular spots and lines under continuous-wave illumination of only a few hundreds of milliwatts. These singular spots and lines evidently originate from the strong SP-induced nonlinearity and photovoltaic effects. We report our work as follows.

## Nonlinear effect

The key sample was an Fe:LN slab coated with ITO thin films on both sides. It was doped with 0.05 wt% Fe_2_O_3_, grown using the conventional Czochralski technique from a congruent melt; the mixing ratio was chosen as Li/Nb = 48.6/51.4. The slab was Z-cut, as-grown, and of dimensions 1.0 × 10.0 × 10.0 mm^3^ with a pair of optically polished opposite surfaces (10.0 × 10.0 mm^2^). The LN slab was coated with 150-nm thick ITO films on both polished surfaces using magnetron sputtering deposition. Several Z-cut ITO Fe:LN slabs were prepared, to ensure the reproducibility of the findings. For comprehensive and precise conclusions, only results from one of the composite slabs are described in this paper.

As discussed in our earlier publications^13^, 2DEG with an electron density comparable to that of noble metals (e.g., silver and gold) can be formed at the ITO/LN interfaces through depolarization of the self-spontaneous polarization field at LN surfaces. Visible SPs are supported in the 2DEG at the interfaces via mediation of the phase gratings present in the photorefractive Fe:LN crystals. Because SPs can confine optical energy within a subwavelength range, the electric field magnitude can be considerably enhanced^1,2^. Consequently, the nonlinearity can be dramatically increased^9,15^. Generally, nonlinear polarization can be expressed as^16,17^

1 $$ \begin{aligned} p_{\mu }^{\omega } & = p_{\mu }^{0} + \varepsilon_{0} \chi_{\mu \alpha } E_{\alpha }^{\omega } + \varepsilon_{0} \chi_{\mu \alpha \beta } \nabla_{\beta } E_{\alpha }^{\omega } + \varepsilon_{0} \chi_{\mu \alpha \beta } E_{\alpha }^{{\omega_{1} }} E_{\beta }^{{\omega_{2} }} \\ & \quad + \varepsilon_{0} \chi_{\mu \alpha \beta \gamma } E_{\alpha }^{{\omega_{1} }} E_{\beta }^{{\omega_{2} }} E_{\gamma }^{{\omega_{3} }} + \varepsilon_{0} \chi_{\mu \alpha \beta } E_{\alpha }^{{\omega_{1} }} B_{\beta }^{{\omega_{2} }} \\ & \quad + \varepsilon_{0} \chi_{\mu \alpha \beta \gamma } E_{\alpha }^{{\omega_{1} }} B_{\beta }^{{\omega_{2} }} B_{\gamma }^{{\omega_{3} }} + \cdots \\ \end{aligned} $$

Note: $\chi_{\mu \alpha \beta }$ and $\chi_{\mu \alpha \beta \gamma }$ are general forms of nonlinear susceptibilities. Although we use the same $\chi_{\mu \alpha \beta }$ for Terms 3, 4, and 6, these may take very different values and even have different dimensions, because they illustrate very different physical processes. The same is true for $\chi_{\mu \alpha \beta \gamma }$. For example, in Term 3, $\chi_{\mu \alpha \beta }$ denotes the contribution of the electric field gradient to the total polarization. In Term 4, the apparently identical coefficient illustrates the dependence on two electric fields, *E*_1_ and *E*_2_, generally with different frequencies, including DC field with zero frequency. Here, a DC spontaneous field is present at the ITO/LN interface; this is rapidly depolarized via electrostatic screening. Consequently, the field gradient is also large though limited to nanometer scales.

Taking a closer look at the ITO/LN interfaces, the spontaneous polarization field is ~ $4.0 \times 10^{10} \;{\text{V/m}}$^18^ and produces a strong photovoltaic effect^19^. Such a high electric field closely resembles the internal atomic field which binds condensed materials. As mentioned in the classical textbooks of optical nonlinearity^16,17^, the hydrogen atomic radius A_0_ = 0.05 nm; therefore, the characteristic atomic electric field is ~ $E_{{{\text{at}}}} = \left( {m^{2} e^{2} } \right)/\left( {4\pi \varepsilon_{0} \hbar^{4} } \right) = 6 \times 10^{11} \;{\text{V/m}}$.

Based on Eq. (1), when a DC field is comparable to the characteristic atomic field, the strength of the second nonlinear effect is comparable to the linear one, and the strength of the third nonlinear effect is comparable to the second one. Using this simple estimation of the nonlinear effect produced by the depolarization field, as well as the large electric field gradient of the nonlinear polarization, we expect interesting phenomena to occur near the ITO/LN interfaces.

## Experimental results

Recently, we observed exotic phenomena at the interfaces; however, we lacked an in-depth understanding of the nonlinearity, as illustrated above. These observations include (1) a transmission valley as low as 10%^20^, (2) a remarkable very first reflection diminishing/enhancement and super high exponential gain coefficients^21^, and (3) an apparent singularity at the interfaces. The first two findings show that within the half-wavelength range, strong energy couplings are generated far from the range of conventional photo-refractivity^22^. Thus, we expect that the interfaces play a key role in the observations. In fact, 2DEGs were found broadly at interfaces of two materials^23,24^. These were treated theoretically using both classical frames^25^ and quantum approaches^26^. The surfaces of the LN crystals were treated using the ab initio perspective, to identify exotic properties not visible in their bulk forms^27,28^. Super-high nonlinear coefficients were observed in ITO in the epsilon-near-zero regime^29^. Here, the connections between the interfacial 2DEG and high nonlinear effect may help to indicate innovative concepts that will facilitate compact photonics and spintronic devices.

**Figure 1:**
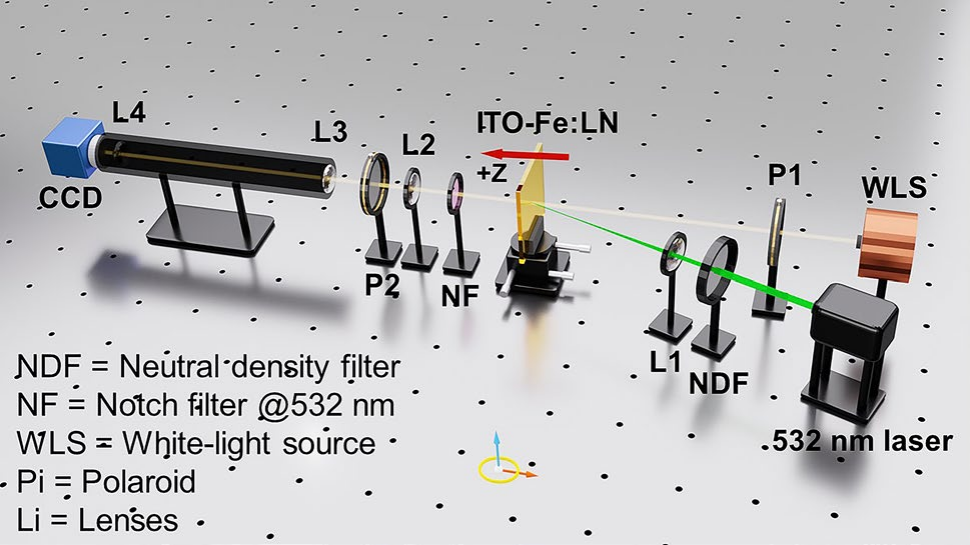
Schematic of experimental setup for monitoring singular spots and lines formed on the two ITO/LN interfaces. The 532 nm (green) laser provided illumination of varying incident power using a neutral density filter. White light was collimated and then directed onto the ITO/LN slab. The notch filter at 532 nm was placed behind the ITO/LN slab, to prevent the green light from arriving at the microscopic system indicated with the two lenses. The charge-coupled-device (CCD) camera recorded the images from the microscopic system.

Regarding singularity, there is no physical explanation given previously, not mentioning real-time dynamics. Here, we apply the clarified nonlinear picture to investigate the phenomena occurring at ITO/LN interfaces. To this end, we constructed a real-time cross-polarization microscopic monitoring system, which can elucidate the phenomena occurring under laser beam illumination (see Fig. 1). Interestingly, we see not only singular spots but also singular lines emerging with more striking features and dynamics. Advancing a large step further, we observed two groups of singular spots and lines that appeared randomly: (1) spots alone and (2) spots and lines. These two groups were associated with two distinct ± Z ITO/LN interfaces. Here, we proceed to a detailed description of the findings.

The real-time cross-polarization microscope setup is shown schematically in Fig. 1. A p-polarization (along the horizontal direction) laser beam of 532 nm (Changchun New Industries, MSL-FN-532) was illuminated onto an ITO/LN slab of varying diameter (0.25–1.5 mm), depending on the focusing condition. The white source of the spectrometer (Ocean Optics, HR4000CG-UV-NIR) was collimated and directed onto the same area; however, it used a much larger diameter, to obtain a broader view.

To demonstrate that the formation of the singular spots and lines was reproducible, we performed experiments using a translation stage to shift the illumination spots, as shown in Fig. 1. When the ± Z-faces of the ITO-coated Fe:LN slab were illuminated with a focused laser beam (0.25 mm diameter, 60.0 mW power), numerous singular spots emerged within a few seconds, in addition to the lines. Using the cross-polarization microscope component (left-hand side of Fig. 1), we can determine whether the singular spots were formed at one of the two ITO/LN interfaces. Because the LN slab was 1.0 mm thick, when we placed the composite slab onto the platform, we could tune the microscope until the singular spots and lines were clearly visible. Then, when we turned the slab backward, the singular spots were blurred completely. Next, we continued to tune the microscope and obtained a clear picture for the second time. This suggests that singular spots and lines were formed on either the + Z-face or the –Z face sides.

**Figure 2:**
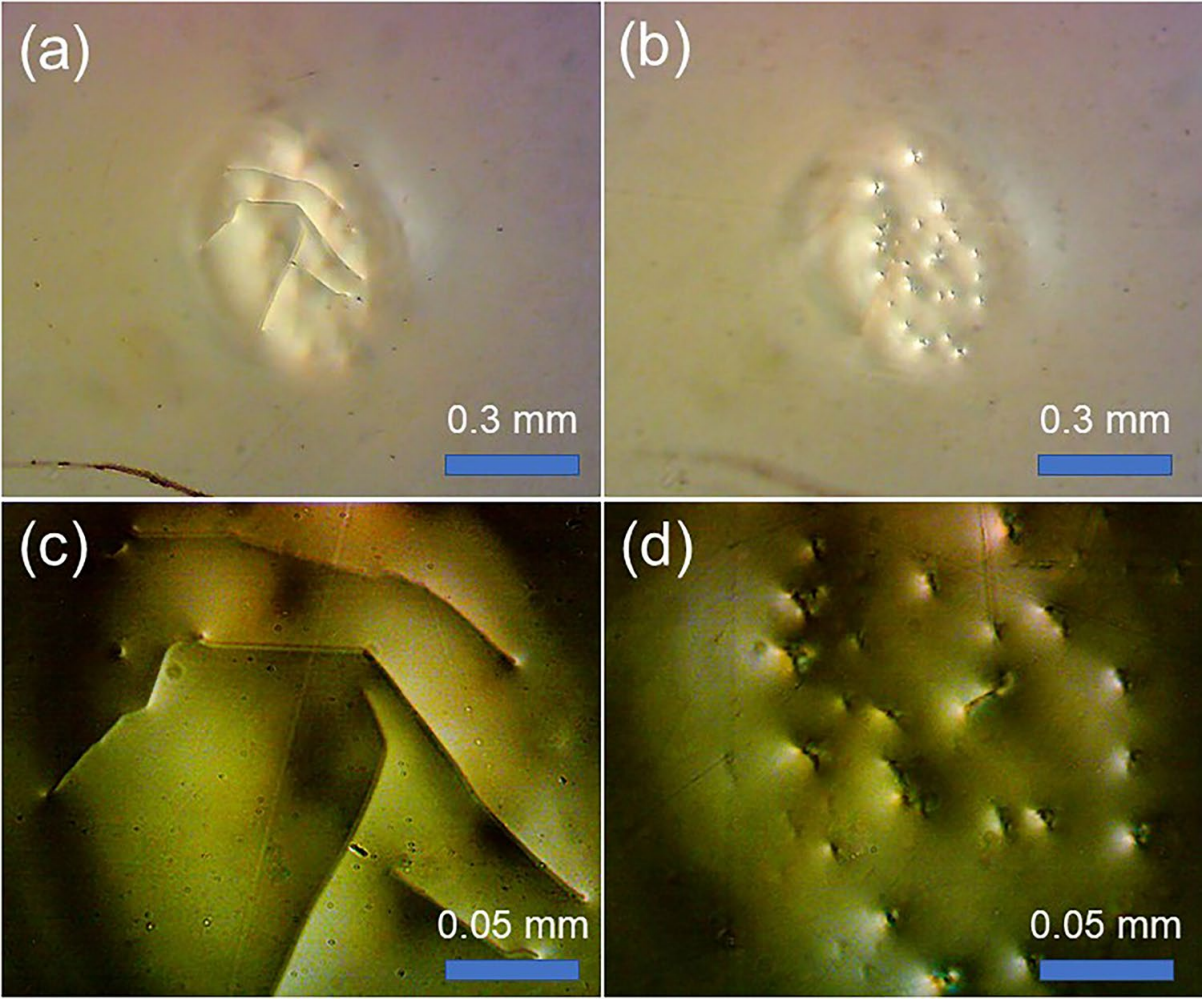
Micrographs taken with the microscopic system: (**a**,**b**) are photographs obtained by focusing on the + and –Z ITO/LN interfaces, respectively, using a 4 × objective; (**c**,**d**) are photographs obtained with a 20 × objective on the + and –Z ITO/LN interfaces.

Focusing on the + Z ITO/LN interface, both singular spots and lines were observed (Fig. 2a,c). Interestingly, the singular spots emerged suddenly, whereas the lines were formed much more slowly and evolved gradually from one spot (i.e., not following a straight line), often terminating at another spot. Refer to the Supplementary Information for the dynamic processes of points and lines evolution in Supplementary Video S1. In contrast, when we focused on the –Z ITO/LN interface side, only singular spots were observed (Fig. 2b,d), even after a considerable period of time (30 min) had elapsed. Regarding the singular spots and lines on the two distinct interfaces, we can obtain some indications from the bottom portion of the photographs shown in Fig. 2a,b. The curve-shaped defect is clearly visible in (a) though quite blurred in (b), because the microscope was focused on the two ITO/LN interfaces.

We believe that the difference between the ± Z faces is attributable to the pyroelectric and photovoltaic effects at the LN surface. The slab operates under an open-circuited condition (i.e., the ITO films on the + Z- and –Z-face sides are not connected); hence, owing to the strong pyroelectric and photovoltaic effects, more electrons accumulate near the + Z ITO/LN interface, whereas more holes accumulate at the –Z ITO/LN interface. Because charge carriers’ accumulations at the two interfaces are different greatly, and hence the mobilities of electrons and holes differ at the two interfaces, consequently, the pyroelectric and photovoltaic-induced breakdowns will differ significantly there. For the + Z ITO/LN interface, lateral breakdowns occur more easily, and more breakdowns result in the gradual formation of singular lines. In contrast, lateral breakdowns do not occur easily; therefore, we cannot observe singular lines near the –Z ITO/LN interface. This will be clarified after discussing the SP-strengthened nonlinearity.

**Figure 3:**
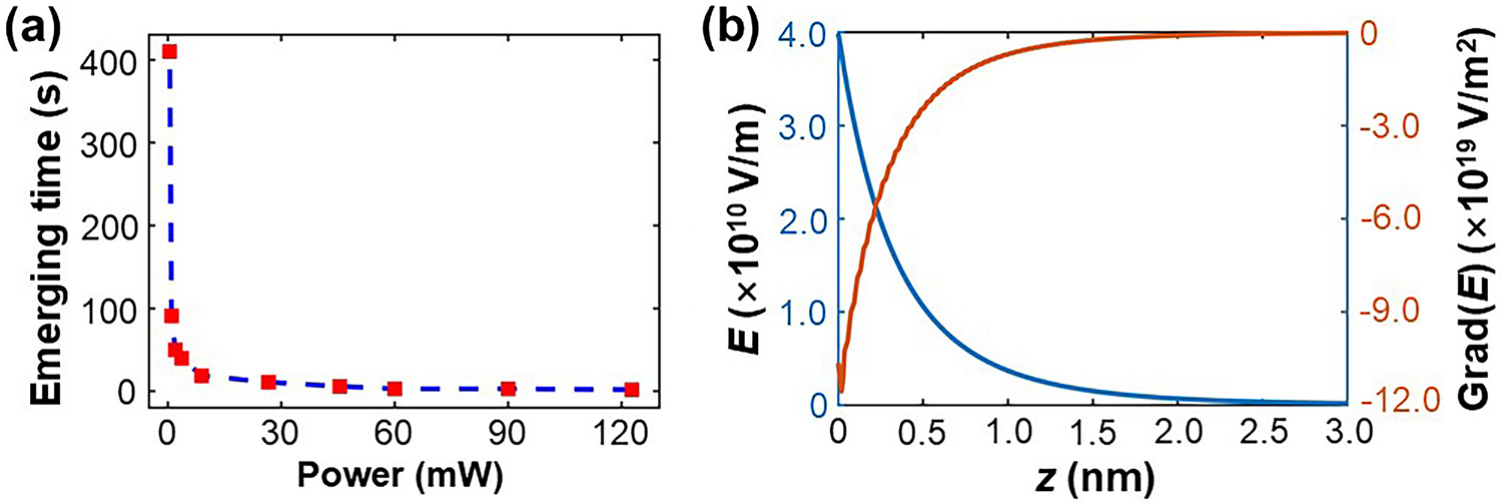
(**a**) The time of the first singular spot’s appearance versus the input power. (**b**) The electrostatic field distribution and its partial derivative distribution (relative to the *z* coordinate) near the ITO/LN interface, with respect to the distance from the interface.

It was found that the emergence time of the first singular spot was highly dependent on intensity. The emergence time is plotted with respect to the varying power in Fig. 3a for a fixed beam diameter of 0.25 mm, where the minimum value of laser beam power is 0.5 mW. Notably, the emergence time increased almost exponentially when the power decreased. Here one may ask a crucial question regarding the slow response time: why the SPPs based spots and lines emerge so slow while a plasmonic processes fall in femtosecond range intrinsically. In fact, the formation of SPPs strengthened slots and lines was dragged down by the response time of photorefractive process and photovoltaic effect. In transition metal doped LN, the response time is in ms to s range, especially under a lower input power. Similarly, the photovoltaic charge accumulation before a beak-downs depends on the input power. It is worth mentioning that similar phenomena can also be observed with lasers beam with different wavelengths, such as the 639 nm laser beam. However, due to the smaller absorption coefficient of the slab, the response time of the first singular spot’s appearance is significantly increased.

**Figure 4:**
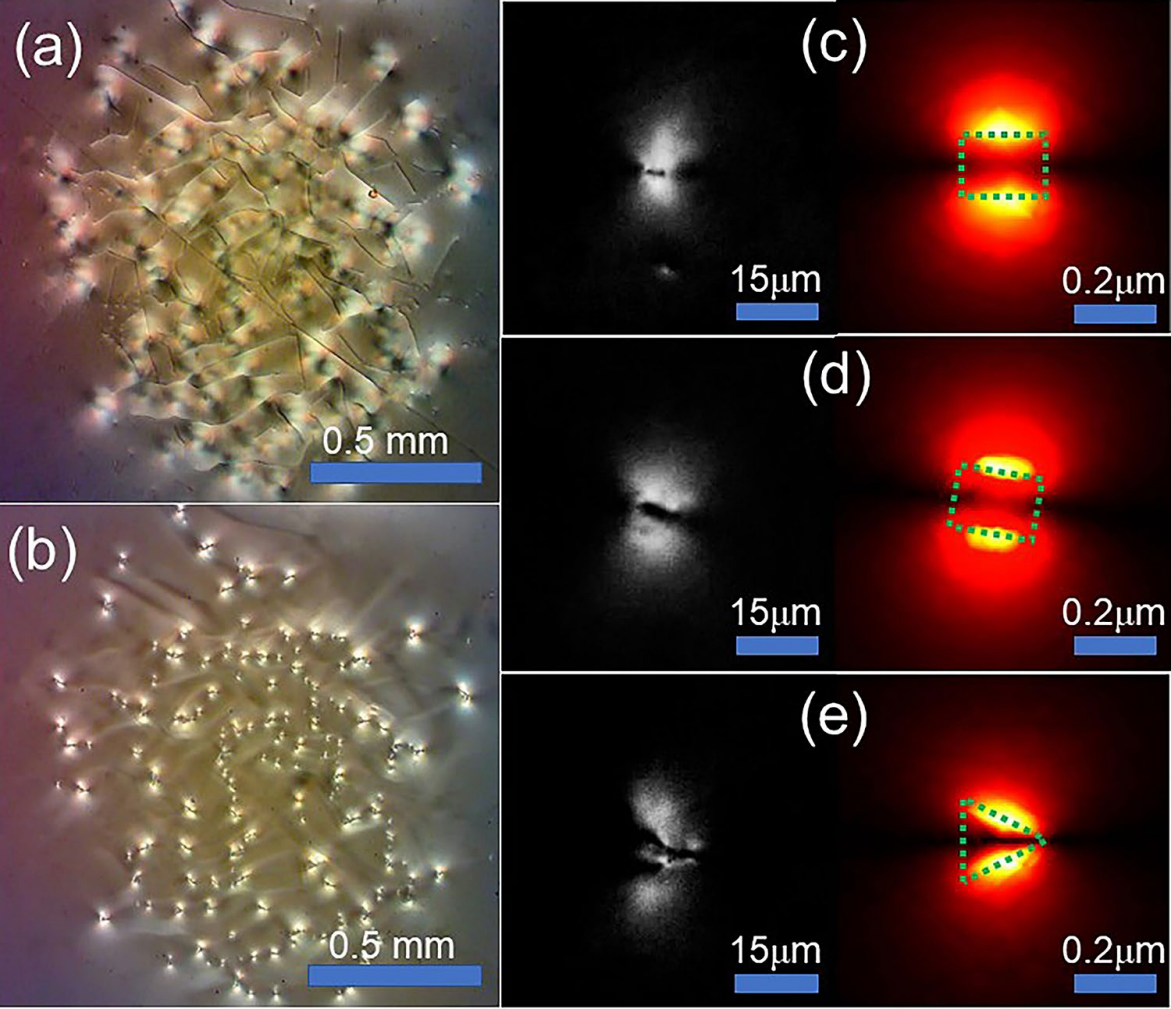
(**a**,**b**) Photographs obtained with a 4 × objective under an unfocused laser beam at the + and –Z ITO/LN interfaces. (**c**)–(**e**) Left: black-and-white micrographs obtained with a 40 × objective, showing details near the singular spots; right: simulation of the field intensity distribution in silver plates with different geometric holes. These holes are indicated by the green dashed lines. The geometries are (**c**) a rectangle of length of 0.2 μm and width 0.15 μm; (**d**) the same rectangle with 10° rotation angle; and (**e**) a trapezoid with 0.16 μm left-hand-side width, 0.02 μm right-hand-side width, and 0.2 μm height.

In fact, the laser beam we used did not need to be focused to observe the aforementioned phenomenon, although it may take longer to become visible. We illuminated the ITO-coated Fe:LN slab using unfocused laser light (1.5 mm beam diameter, 122.7 mW input power). Under a 4 × objective, numerous singular spots and lines were formed (Fig. 4a,b).

To clearly observe the phase changes in the singular spots, we used black and white images with a 40 × microscope objective. In these images, within each singular spot on the -Z-face, two bowtie-like lobes [the left-hand side of Fig. 4c–e] were visible. These singular spots varied slightly under constant input power. However, the seemingly broken spots could be eliminated by heating the sample above 200 °C for over 1 h.

To investigate the mechanism behind the formation of singular spots and lines at the two ITO/LN interfaces, we examined the depolarization field and electrostatic field gradient. As illustrated in^13^, the polarization field screening was confined within the nanometer range. The polarization field penetrating the screening layer was calculated and is shown in Fig. 3b. An electric field as high as 10^10^ V/m was present within the nanometer range. Because such a large polarization field was screened within this range, the field gradient was very large, as shown in Fig. 3b. Returning to Eq. (1), the DC field and field gradient can enhance the nonlinear effect in the nanometer range, combining to form the remarkable SPs and subsequent optical-state-intensity increase.

After verifying that the singular spots were reproducibly formed near one of the interfaces, it was suggested that 2DEG was responsible for their formation, implying that visible SPs are supported at the interfaces. Defects at the interface (e.g., defects in polishing and deposition) considerably influence the excitation and propagation of SPs. A strong photovoltaic effect is known to occur in the Fe:LN slab; this can cause frequent local breakdowns. It is also known that ITO coating can significantly mitigate these frequent breakdowns, although it cannot eliminate them. Once the photovoltaic-induced breakdown occurs, the local refraction index can be altered, generating a defect in the SP excitation environment. Therefore, local breakdowns can serve as spontaneous defects in the SP excitation. This is consistent with the abrupt emergence of singular spots and accords well with the aforementioned emergence time’s dependence on the input power, where the photovoltaic field depends on the illumination intensity. Therefore, the photovoltaic charge accumulation before breakdown actually depends upon intensity. With a fixed focusing spot size, the weaker the incident power, the longer the waiting time before breakdown; hence, the longer the emergence time of the singular spots and lines. As discussed in brief earlier, this also accords with the difference in singular spot and line formation at the ± Z ITO/LN interfaces, owing to the different charge accumulation conditions.

To simulate the formation of these singular spots and lines, we must consider the propagation and localization of SPs. Near the ITO/LN interface, 2DEG is formed with sufficient density to support visible SPs. Under illumination of the ITO-coated Fe:LN, phase gratings can be written owing to multiple reflections. Once phase gratings are written, SPs can be excited more efficiently, in addition to the SPs excitation due to unevenness of the ITO/LN interfaces. Consequently, the electromagnetic field can be significantly strengthened. Moreover, if the refractive index of the surrounding medium is changed, the propagation of SPs can be altered accordingly. If a sufficiently strong change occurs, SP localization can arise. When localization occurs, the electromagnetic field can be significantly intensified. When a photovoltaic breakdown occurs, the SP propagation can be interrupted. This can be treated equivalently in the following cases:

The cases can be regarded as the influence of geometric holes on the distribution of the SP excitation field intensity in the metal plate^30,31^. Because the electron density is comparable to that of noble metals, silver can be used to simulate the case in our composite system. We used the CST Studio Suite to simulate the field intensity distribution of silver thin films surrounding air with different geometric holes under vertically polarized plane wave illumination. When the light’s polarization direction is parallel to the edge of the hole, the SPs will not be excited. The selective excitation of SPs results in a specific spatial distribution of field intensity [right-hand side of Fig. 4c–e]. The simulation results are consistent with the corresponding black and white images on the left-hand side. In addition, this explains why the lobes near all spots are aligned in the same direction [see Fig. 4b]; for line-shaped singular defects, these can be regarded as numerous localized SP spots connected to each other.

## Phase change for spots

To validate the physical picture, we verified the specific phase change for those singular spots. Near one singular spot, there is a π phase shift from the brightest part to the darkest part. Conservatively speaking, such a phase change was resulted from a physical process within half a wavelength, owing to the evanescent nature of SP waves. For 532 nm light (the most sensitive to the eyes), such as half-wavelength is $l = 0.535/\left( {2.2 \times 2} \right) = 0.121\;\upmu {\text{m}}$. The incident beam diameter was $d = 1.5\;{\text{mm}}$ and the input power was $P = 122.7\;{\text{mW}}$. The approximate SP field intensity was $I = P/(d/l)$. Considering the polarization field *E*(*z*), the third-rank nonlinear effect producing the refractive index change can be expressed as

2 $$ \Delta n(z) = n_{2} \times I = \frac{{{\text{Re}} \chi^{(3)} \times E(z)}}{{2n_{0}^{2} \varepsilon_{0} c}} \times I, $$

where *n*_2_ is the Kerr coefficient, *n*_0_ is the refractive index of LN, *c* is the speed of light, and $\varepsilon_{0}$ is the vacuum permittivity. ${\text{Re}} \chi^{(3)}$ is the real part of the third nonlinear polarizability of LN. The integral of the refractive index change over half a wavelength generates a π-phase shift of

3 $$ \pi = \frac{2\pi }{\lambda }\int\limits_{0}^{{0.121 \times 10^{ - 6} }} {\Delta ndz} . $$

After substituting Eq. (2) into Eq. (3) and integrating, we obtain the third-order nonlinear polarizability ${\text{Re}} \chi^{(3)} = 6.12 \times 10^{ - 19} \;{\text{m}}^{2} /{\text{V}}^{2}$. This value exceeds that previously reported^32^ though is close to the reported number. In fact, we neglected contributions from other nonlinear terms in Eq. (1) under the change in refractive index, including the aforementioned large contribution of the electric field gradient to the polarization. This may partly explain the exaggerated nonlinear polarizability. This simple estimation indicates the large refractive index change, which is much higher than that observed conventionally. The π phase shift within half a wavelength is unprecedented. In electro-optics applications, the π phase shift is crucial for many applications. Here, in such interfaces, a π phase shift is realized within the nanometer range. This demonstrates the potential of this work’s findings for nano-photonics applications.

Here, we only partly considered the contribution of the third-rank nonlinear effect. In fact, Eq. (1) shows that many other nonlinear effects can manifest themselves in the ITO/LN composite system, owing to the special combination of properties typically absent from bulk states; these properties are as follows: (1) the 2DEG is as dense as that observed in noble metals, (2) the DC field is as high as that observed in the atomic fields which bind the materials together, (3) the electric field has a very large gradient, and (4) reconfigurable phase gratings are present near the ITO/LN interface and in the body. Research work in these areas is underway.

It deserves to be mentioned here that the huge electric field and its gradients are universal at ferroelectric surfaces and interfaces with other nonpolar oxides. The huge electric field and electric field gradient, though only existing in a nanometer thickness, can strengthen the nonlinear effects by as many as 7–10 orders of magnitudes, this alone can be highly interesting for interface based devices.

Regarding the spots and lines seen with microscopy, the phase jump is the result of an interface, much shorter than half a wavelength, this should be quite interesting for understanding the physics near the interface. More study along this line by readership may be proven productive.

## Conclusions

To conclude, singular spots and lines formed at ITO/LN interfaces seem to originate from SPs; they produce a strong nonlinearity, attributable to both the large depolarization field and the large electric field gradient. The difference between the two interfaces is attributable to the photovoltaic-induced breakdowns. The nonlinear effects produce a large refractive index change within a half-wavelength range. The intriguing findings at the two ITO/LN interfaces deserve further investigation, and they may prove very promising for the design and implementation of visible, compact photonic devices and sensor systems.

## Supplementary Information

Supplementary Legends.

Supplementary Video 1.

## Data Availability

The data that support the findings of this study are available from the corresponding author upon reasonable request.
