# Supplementary material for: Surface plasmon induced spot and line formation at interfaces of ITO coated LiNbO3 slabs and gigantic nonlinearity
Source: Sci Rep. 2021 Oct 5;11:19790. doi: 10.1038/s41598-021-99270-4 (PMC8492684; doi:10.1038/s41598-021-99270-4)
Supplement: Supplementary file 1 — Supplementary Legends. [file 41598_2021_99270_MOESM1_ESM.docx]

**Supplementary Information**

**Surface plasmon induced spot and line formation at interfaces of ITO coated LiNbO3 slabs and gigantic nonlinearity**

Zuoren Xiong^1,2^, Xinyan Ma^1,3^, Yanbo Pei^1,3^, Yingbin Zhang^1,2^ & Hua Zhao^1,3,*^

^1^Institute of Modern Optics, School of Physics, Harbin Institute of Technology, Harbin, 150001, China

^2^Key Laboratory of Micro-Optics and Photonics Technology of Heilongjiang Province, Harbin, 150001, China

^3^Key Laboratory of Micro-Nano Optoelectronic Information System, Ministry of Industry and Information Technology, Harbin 150001, China

***[zhaohuaz@hit.edu.cn](mailto:zhaohuaz@hit.edu.cn)

**Video S1: The dynamic processes of points and lines evolution.**

Video S1 shows the emergence of singular points and lines at the +Z ITO/LN interface. The ITO-coated Fe:LN slab was illuminated with a focused laser beam (0.25 mm diameter, 60.0 mW power). In this case, both singular spots and lines were observed.
